# The Clinical Utility of Pulmonary Function Tests in the Diagnosis and Characterization of Central Airway Obstruction: A Narrative Review

**DOI:** 10.3390/jcm13216299

**Published:** 2024-10-22

**Authors:** Dimitrios Ampazis, Vasileios Vlachakos, Nektarios Anagnostopoulos, Argyrios Tzouvelekis, Fotios Sampsonas

**Affiliations:** 1 Respiratory Department, Cavan & Monaghan Hospital, HSE/RCSI Hospital Group, H12Y7W1 Cavan, Ireland; dim_ampazis@yahoo.gr; 2 Bioclinic General Hospital of Athens, Henry Dunant Hospital Center, 11526 Athens, Greece; vasvlahakos@gmail.com; 3 Interventional Pulmonology Unit, 1st University Respiratory Department, “Sotiria” Chest Diseases Hospital of Athens, 11527 Athens, Greece; aris.anag@yahoo.gr; 4 Respiratory Department, Patras University Hospital, 26504 Patras, Greece; argyris.tzouvelekis@gmail.com

**Keywords:** central airway obstruction, pulmonary function tests, oscillometry, plethysmograpy, diagnostic utility, bronchoscopy

## Abstract

Central airway obstruction is of major clinical importance since it is a major cause of morbidity and mortality and is usually clinically diagnosed late. Pulmonary function tests, with the recent incorporation of oscillometry, can have a crucial role in earlier diagnosis. In this review, we summarize all recent advances in that view, including the crucial role of oscillometry.

## 1. Introduction

Central airway obstruction (CAO) represents a critical and frequently underdiagnosed condition affecting both adults and children, often leading to significant respiratory compromise. It typically involves an occlusion of more than 50% of the diameter of the trachea, mainstem bronchi, bronchus intermedius, or of a lobar bronchus [1].

CAO can arise from multiple disorders, and it could be congenital or acquired. The obstruction may be fixed or caused by an endoluminal, extraluminal or mixed lesion. Alternatively, dynamic obstruction can result from cartilage or pars membranous flaccidity [2]. The collapsibility index is used to characterize the dynamics of CAO and reflects the difference in airway lumen diameter between inspiration and expiration. Variable CAO is defined by a collapsibility index of greater than 50%, whereas fixed CAO is characterized by an index of less than 50% [3]. Τhe primary approach involves a comprehensive evaluation to determine the cause of the obstruction. Malignant tumors may cause CAO, both from direct extension of the disease or from extrinsic compression. CAO is identified in about one in eight lung cancer patients, with an additional 5% developing CAO during follow-up [4]. Bronchogenic carcinoma, esophageal cancer, and thyroid carcinoma are the malignant tumors most commonly responsible for CAO. Nearly all types of solid tumours have the potential to occasionally metastasize into the bronchial lumen [5]. Numerous non-malignant etiologies can lead to acquired central airway obstruction (CAO), including non-malignant tumors, infectious diseases (which vary by geographic location), autoimmune disorders, iatrogenic factors, and other causes. Acquired non-malignant CAO is most frequently caused by iatrogenic tracheal injury [6]. Congenital CAO is rare in adults and is usually caused by the posterior fusion of the tracheal rings. In children, CAO is frequently associated with congenital cardiovascular abnormalities, such as anomalous bifurcation of the innominate artery, which can cause compression of the tracheal wall [7]. Tracheobronchomalacia may develop along with congenital conditions such as Marfan syndrome, cystic fibrosis and Ehlers-Danlos syndrome [8]. A myriad of benign and malignant diseases is associated with airway obstruction. An excellent list of these diseases can be found in the classical paper by Ernst et al. [9].

## 2. Clinical Presentation and Diagnostic Workup of Patients with CAO

Clinical presentation will depend on the location, extension and degree of obstruction, the speed of progression, and the underlying disease. Nearly half of patients with CAO initially present with dyspnoea as their main symptom [10]. Exertional dyspnoea is the characteristic symptom of laryngotracheal stenosis and is often the primary cause of morbidity and disability [11]. As the occlusion progresses, symptoms such as cough and wheezing will emerge. In cases of laryngeal involvement, clinical examination may reveal hoarseness. Stridor is a sign of severe laryngeal or tracheal obstruction. The patient may complain of recurrent infections and impaired sputum clearance, or haemoptysis. Symptoms typically arise after a moderate obstruction (50–70% reduction in the cross-sectional area), especially during periods of increased airflow, such as in exercise. Sedentary patients often remain asymptomatic at this level of lumen occlusion. However, severe stenosis can cause symptoms even at rest or with minimal exertion, necessitating intervention [1].

Upon suspicion of CAO, the initial investigations include imaging, chest X-Ray (CXR) and computed tomography (CT), blood tests, pulmonary function tests (PFTs), and bronchoscopy. Blood testing usually has non-specific changes but such work-up may be useful in identifying possible underlying conditions [2]. Measurable changes in pulmonary function tests typically occur when the airway obstruction exceeds 80%. Flattening of the inspiratory loop may indicate a variable extra-thoracic obstruction, whereas an intrathoracic obstruction causes flattening of the expiratory loop. A fixed obstruction may result in flattening of both loops. Diagnosis of tracheal tumors through chest radiography occurs in fewer than 30% of patients [12].

CT is the imaging modality of choice, with a sensitivity of 97% [13]. A CT scan is useful for identifying the location, assessing the extent and characteristics of the lesion, determining the degree of stenosis, and evaluating the patency of the distal airway. While CT scans can effectively detect airway obstruction, the accuracy of the examination hinges on the radiologist’s awareness to identify potential endoluminal abnormalities that may be overlooked [14]. A dynamic expiratory CT scan is particularly valuable for diagnosing cases of dynamic obstruction, such as tracheobronchomalacia. The role of MRI in the diagnostic workup of CAO is limited, as it is mainly utilized for assessing the mediastinum, offering detailed images of its structures. Fiberoptic bronchoscopy is the gold standard for evaluating and monitoring endoluminal lesions [15]. Flexible and rigid bronchoscopy are useful in the direct visualization of endoluminal lesions, collecting samples for microbiological and cytologic analyses, and enabling the planning of appropriate endobronchial therapy, if feasible [1].

The aim of this review is to characterize the utility of PFTs tests in patients with central airway obstruction. We will summarize the specificity and sensitivity of spirometry and flow-volume curves (FV), plethysmography and oscillometry in these patients to assist clinicians in identifying and characterizing central airway obstruction when encountered by employing the most appropriate pulmonary function test.

- **Population**: Patients with suspected central airway obstruction
- **Intervention**: PFTS, including spirometry and flow-volume curves, oscillometry and plethysmography
- **Outcome**: To identify the specificity and sensitivity of PFTs in characterizing the severity, and therefore the need of intervention, and location of airway obstruction

## 3. Overview of Terminology and Measurements of Pulmonary Function Tests in Patients with CAO

When approaching CAO with pulmonary function tests [PFTs], it is useful to provide an overview of the definitions for the different measurements and parameters that are commonly used. Most of the parameters are derived from the spirometric measurements. This is expected given that spirometry is the most widely used pulmonary function test and is more easily accessible to clinicians in comparison to other more advanced modalities. However, other techniques and tests have been utilized and relevant data are presented.

The rationale by which the literature data are deployed below is based on two parameters: how frequently a measurement has been utilized and its level of sensitivity and specificity.

The relevant terminology is as following:

### 3.1. Spirometry—Quantitative Criteria

1. FEF50%/FIF50%: ratio of the flow at the mid-point of the forced expiratory maneuver (FEF 50%) to the flow at the mid-point of the forced inspiratory maneuver (FIF 50%) [also denoted as MEF50%/MIF50%];
2. FEV1/PEF: the ratio of forced expiratory volume in the first second (FEV1) to the peak expiratory flow (PEF) [also called Empey index];
3. FEV1/PEFx100: ratio of forced expiratory volume in the first second (FEV1) to the peak expiratory flow (PEF) multiplied by 100 [also called the Expiratory Disproportional Index, a refined version of the Empey index];
4. PEF: peak expiratory flow;
5. PIF: peak inspiratory flow;
6. FIF50%: flow at the mid-point of the forced inspiratory maneuver;
7. FEV1/FEV0.5: ratio of forced expiratory volume in the first second (FEV1) to the forced expiratory effort in 0.5 s;
8. MVV/FEV1: ratio of the maximum voluntary ventilation to the forced expiratory volume in the first second (FEV1);
9. SVC—FVC: difference between slow vital capacity and forced vital capacity.

### 3.2. Spirometry—Qualitative Criteria

Further to the above quantitative criteria, spirometry can also provide qualitative criteria through the evaluation of the shape and other features of the inspiratory and expiratory flow-volume loops.

The qualitative criteria utilize characteristics of the flow-volume loops that can be visualized, and they usually focus on the following features:

1. Inspiratory curve truncation or flattening [plateau];
2. Expiratory curve truncation or flattening [plateau];
3. Inspiratory and expiratory curve truncation or flattening [plateau];
4. Inspiratory or/and expiratory biphasic phase;
5. Inspiratory or/and expiratory oscillations [saw-tooth pattern].

The aforementioned spirometric quantitative and qualitative criteria, albeit the most common, are not the only ones used.

### 3.3. Body Box Plethysmography

Other modalities have also been used in assessing the central airways stenosis.

Whole body [body box] plethysmography has been utilized to provide:

1. Raw: airway resistance,
2. sGaw: specific airway conductance.

### 3.4. Volume-Pressure Curve and Compliance

Another measurement is that of compliance, utilizing the pressure-volume curve.

### 3.5. Oscillometry

In addition to the above methods, oscillometry can also provide information regarding the central airways making use of:

1. Rrs: resistance of the respiratory system [measurements at 5 and 20 Hz];
2. Xrs: reactance of the respiratory system [measurements at 5 Hz];
3. Zrs: impedance of the respiratory system;
4. Rf: resonance frequency [or Fres];
5. ALX: low-frequency reactance area.

## 4. Relevant Clinical Questions

All the above measurements and parameters seek to answer different questions in relation to the nature and severity of the CAO. Clinical scenarios may raise several concerns regarding the suspected stenosis and thus, the interest is primarily focused on:

1. The level of the stenosis [intrathoracic or extrathoracic];
2. The wall region involved, when the trachea or main airways are investigated [membranous/posterior wall vs. cartilaginous wall];
3. The pattern of the airway caliber changes [fixed or variable stenosis];
4. The severity of the stenosis [usually expressed in percentages of the normal diameter of the airway];
5. The improvement of the stenosis after an intervention;
6. The coexistence of central and peripheral airway obstruction.

## 5. An Overview of the Sensitivity and Specificity of PFTs of the Spirometric Findings

As mentioned earlier, the most common measurements are derived from spirometry. Spirometry is based on forceful inspiratory and expiratory maneuvers that can be technically challenging for both the patient and the clinician. This can strongly affect the quality of the outcome. It is advisable that at least 3 efforts have to be performed, and the flow-volume loops are expected to have repeatable patterns at a maximal inspiratory and expiratory effort. By the end of a spirometric effort, apart from the flow-volume curves, measurements—the quantitative parameters—are also available. The sensitivity and specificity are not equally robust between the quantitative and the qualitative criteria.

In their study, Raposo et al. found that when the quantitative criteria were used, both the sensitivity and specificity were higher, while with the use of qualitative/morphological criteria the sensitivity is low, but the specificity is higher [16].

Regarding the qualitative [visual] criteria [shape/features of the flow-volume curves], the literature provides variable sensitivities [from as low as 5.5% up to 100%] and specificities [78% to 93.6%] [17,18,19,20,21], making their usefulness as screening tool for CAO questionable. A quite characteristic example is the study by Modrykamien et al., which presents a significantly low sensitivity and positive predictive value, at 5.5% and 6.8%, respectively, while the specificity and negative predictive value were 93.8% and 92.3%, respectively [22].

There are not many studies that try to approach the qualitative and quantitative spirometric measurements for CAO, and the cohorts of patients vary significantly with either homogeneous characteristics [e.g., extraluminal, extrathoracic obstruction, where high sensitivities and specificities are achieved] or heterogeneous characteristics [populations with different types of CAO, where variable sensitivities and specificities are reached]. This explains, to some extent, the significant variability of both sensitivity and specificity provided by different research efforts.

Table 1 summarizes the studies that have provided details in relation to sensitivity and specificity regarding qualitative and quantitative criteria.

The literature findings concur that the airflow limitation, due to CAO, needs to be quite significant so as to be accurately diagnosed and characterized by the PFTs and become clinically correlated to the symptoms [17]. In a model study by Brouns et al., flow patterns and pressure drops were assessed over tracheal stenoses. This became feasible through an artificial model, derived from multislice CT images obtained from healthy men, that replicated a three-dimensional upper airway. The measurements showed that a decrease in the airway caliber of more than 70% is required for this to be considered significant. The model measurements were specifically focused on the subglottic region of the trachea [28].

The above finding, correlating the obstruction—above 70%—with the airflow limitation and the airway resistance, was confirmed in a model study by Ellingsen and Holmedahl. In their model, the flow resistance increased significantly when the large airway cross-sectional area was reduced, due to excessive dynamic airway collapse (EDAC), by more than 70% for an obstruction length of 3 cm [29]. However, it is interesting that, when the measurements were reproduced on patients for similar degrees of CAO due to EDAC, they were clinically significant on cough maneuvers only, but not on forced expiration, and thus not reflected in PFT values [29].

It is worth noting that the literature agrees that bronchoscopic significant EDAC is considered a reduction in the main airway diameter of above 50% but this is not necessarily reflected in the PFT measurements. In conjunction with the above, it has been shown that the diameter of the large airways, the trachea in particular, has to decrease below 8 mm for the CAO to be detectable and clinically pronounced [30,31,32].

## 6. Spirometric Quantitative Criteria—FEF50%/FIF50%:

The most commonly used spirometric quantitative criterion is the FEF50%/FIF50%. The values have been correlated with different types of CAO [33,34,35,36,37,38,39,40]:

- 0.85–1 can be indicative of fixed airway obstruction [intra or extra thoracic];
- ≥1 indicates extrathoracic obstruction;
- ≥2.2 is in keeping with variable extrathoracic obstruction;
- 0.32 demonstrates variable intrathoracic obstruction.

The above values were originally defined as indicative of the relevant patterns by the well-known studies of Miller and Hyat [30,41]. More recent studies, however, have challenged the accuracy of these indices. In their study, Sterner et al. found that a mid-flow ratio of 2.2 was a poor predictor of variable extrathoracic obstruction. According to this study, only 60% of the studied population had reproducible inspiratory flow-volume loops affected by the obstruction, and the percentage was even lower, down to 28%, when the best inspiratory curve was selected [18]. Further to that, the most common extrathoracic obstruction detected was functional in nature (vocal cord dysfunction) while the anatomical abnormalities were far less.

Garcia-Pachon et al. found that, despite being among the most sensitive indices in detecting CAO, the mid-flow ratio’s sensitivity decreases when chronic airflow limitation is present due to peripheral airway disease [27]. Similar conclusions, regarding the lower sensitivity and specificity of the spirometric values in CAO, were drawn by Handa et al. In their study, which utilized spirometric and oscillometric measurements for CAO, although the spirometric values could reflect post-bronchoscopic intervention improvements in the management of CAO, they were not able to specify if the obstructive element—before the intervention—was purely due to the CAO or because of coexisting peripheral airway disease [3].

Another parameter that can affect the mid-flow ratio is the quality of the initial phase of the curve, when it is performed to capture maximal flows. It is primarily the initial inspiratory/expiratory phase that would reveal the element of the central obstruction. If the initial peak effort is not well performed, the flow drop, which will inevitably occur later on in the maneuver due to the contribution of the peripheral airways, will mask any central obstruction [42].

Despite the arguments in relation to its sensitivity and specificity in detecting the size and location of CAO, FEF50%/FIF50% is still utilized in that view. It may raise the suspicion for airway obstruction within the different levels of the large airways—apart from the mid third of the trachea—and therefore alert clinicians to pursue further investigations. This is well supported in the study by Raposo et al., who suggest that FEF50%/FIF50% ≥ 1 correlates well with the presence of CAO at any level of the airway, excluding the mid-trachea, in 83% of patients [16].

## 7. Spirometric Quantitative Criteria—Empey Index and Expiratory Disproportional Index [EDI]

The next most common quantitative criterion is the Empey index and its refined version, the expiratory disproportion index [16,21,34,43,44,45]. The FEV1/PEF ratio was introduced by Empey as a simple yet sensitive measurement for CAO and had set, as the cut-off point, a value of 10 mL/L/min, above which CAO is very likely [44].

Rotman et al. agreed with the utility of the FEV1/PEF regarding CAO [34], while Miller et al. recommended that values above 8 mL/L/min provide a more sensitive cut-off point to identify CAO when provoked by external compression, from goiter in particular [21].

The study by Raposo et al. confirmed the usefulness of the FEV1/PEF ratio for CAO. Their findings suggested that it is sensitive regarding the detection of obstruction in the lower 1/3 of the trachea, without, however, being able to discriminate between intraluminal or extraluminal obstruction [16]. A similar positive correlation of the Empey index with CAO—concerning tracheal stenosis in particular—was found by Linhas et al., in a study evaluating spirometric and oscillometric indices. Their findings regarding the FEV1/PEF ratio suggest that it correlates well with the degree of tracheal stenosis [46].

The sensitivity and specificity, however, remain to be further clarified when peripheral airway obstruction coexists [27]. The refined version of the Empey index is the expiratory disproportion index, which is calculated as FEV1 [L]/PEF [L/s] × 100. Nouraei et al. recognized its value as a sensitive and specific measurement which can indicate severe anatomic obstruction, most likely grade 3 stenosis or laryngeal obstruction, when its value is above 75 [47]. However, this index cannot differentiate between fixed and variable obstruction or between intrathoracic and extrathoracic obstruction. Nevertheless, it is very useful in reliably identifying upper airway obstruction and thus they promoted its use as a screening tool for populations that are at increased risk for CAO. Their recommendation suggests that values above 50 should raise the suspicion of CAO and generate further investigations [47].

In another study, Schuering et al. showed that the EDI can discriminate subglottic stenosis from asthma, and they recommended, as a useful cut-off point, values above 48. However, this study only focused on benign stenosis. Furthermore, it commented that lower sensitivities were achieved when peripheral airway disease coexisted [24]. Calamari et al. utilized the EDI for the detection of upper airway obstruction in individuals with a BMI above 30, and suggested that its use might be valuable, when values were above 50, to raise the suspicion of functional obstruction due to increased BMI [25].

In a study by Ntouniadakis et al., the EDI showed acceptable sensitivity, when the values were above 39, in detecting subglottic stenosis vs. asthma or COPD. Their findings advocate that the EDI can raise the suspicion of extrathoracic airway obstruction and thus generate further investigations [26].

## 8. Spirometric Quantitative Criteria—PIF and PEF

The role of PEF and PIF has also been commented on in the literature, but in a less systematic way.

PIF of less than 100 L/min may suggest CAO with extrathoracic element, being however less specific primarily in view of the technical challenge regarding the optimal inspiratory maneuver, which might be difficult for the patient to perform [34].

The role of PEF in CAO has also been studied as a useful and simple tool in detecting the relevant defects. Miller et al. have shown that, at high lung volumes, PEF is generally decreased in both intrathoracic and extrathoracic lesions. In variable CAO however, PEF might be normal with vocal cord paralysis being a characteristic example [21]. In their study, Linhas et al. tried to assess spirometric and oscillometric findings before and after bronchoscopic intervention for confirmed tracheal stenosis. The results suggested that that PEF values are inversely correlated with the length of stenosis [46]. Nuraei et al. conducted a study focusing on laryngotracheal stenosis cases that underwent laryngotracheoplasty with concomitant evaluation of preoperative spirometric parameters and intraoperative changes in compliance. Their findings suggested that the reduction of PEF in laryngotracheal stenosis was only moderately correlated with the subglottic stenosis [48].

Like other spirometric measurements, the sensitivity and specificity of the PEF in CAO might be lower if peripheral airway diseases coexist [3,27,49]. However, Yasuo et al. investigated differences in spirometric and oscillometric values between CAO and COPD, finding that patients with CAO showed a significant reduction in PEF and that this was also correlated with most of the oscillometric parameters [50].

## 9. Spirometric Quantitative Criteria—Other Measurements

Regarding other measurements derived from spirometry, the FIF50%, FEV1/FEV0.5, MVV/FEV1 and SVC-FVC have also been used. The relevant values that suggest CAO are FIF50% < 100 mL/min, FEV1/FEV0.5 ≥ 1.5, MVV/FEV1 < 25 [34,44,51], and SVC-FVC > 100 mL. The above indices are not extensively utilized and thus data in the literature are less well described.

Garcia-Pachon et al. tried to compare the utility of several indices for patients with upper airway obstruction [UAO] with or without chronic airflow limitation [CAL]. They suggested that the most specific index for identifying UAO is the MVV/FEV1, especially when peripheral airflow limitation coexists [27].

A difference of more than 100 mL between SVC and FVC has been used to identify an element of variable CAO, primarily due to EDAC. This however, has variable sensitivity and specificity [32].

## 10. Spirometric Qualitative Criteria

The PFTs, as already demonstrated, can provide measurements and values that quantify the phenomena. It is however significantly useful when the PFT modalities can also provide morphological data. The shapes and features of the flow-volume loops in spirometry can be utilized in that view. The inspiratory and expiratory curves have traditionally been used to explain how the airways behave in different disease patterns.

In CAO, the most common features are the following [17]:

- Plateau of the curve of the forced inspiratory flow, with or without forced expiratory plateau, suggesting a variable central or upper extrathoracic obstruction;
- Plateau of the curve of the forced expiratory flow, along with lack of forced inspiratory plateau, suggesting a variable intrathoracic obstruction;
- Plateau, at a similar flow in both the inspiratory and expiratory phase, suggesting a fixed central or upper airway obstruction;
- Flow oscillations [saw-tooth pattern], during either the inspiratory or expiratory phase, probably representing a mechanical instability of the wall, provided that the maneuver has appropriately been performed.

**Figure 1:**
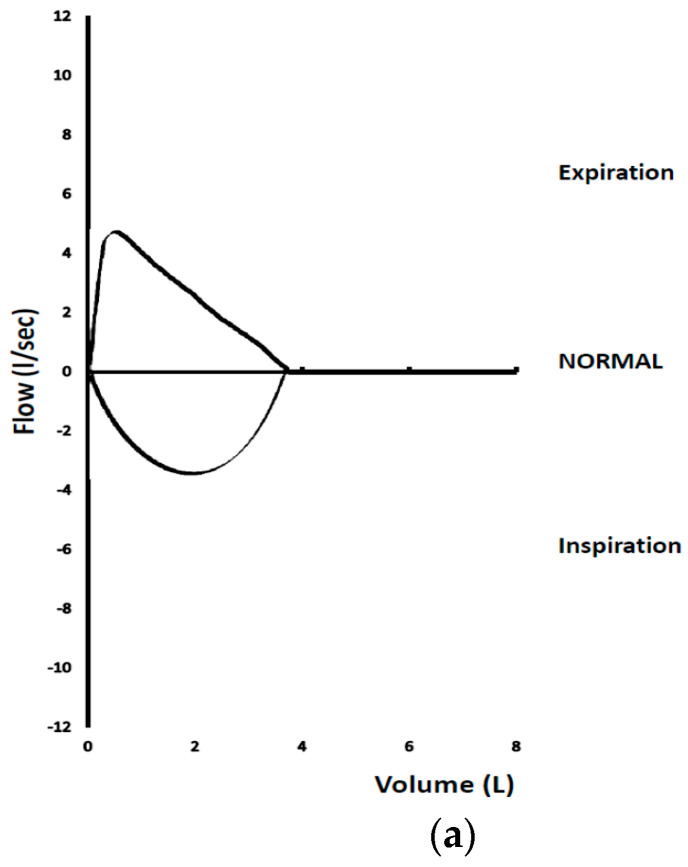

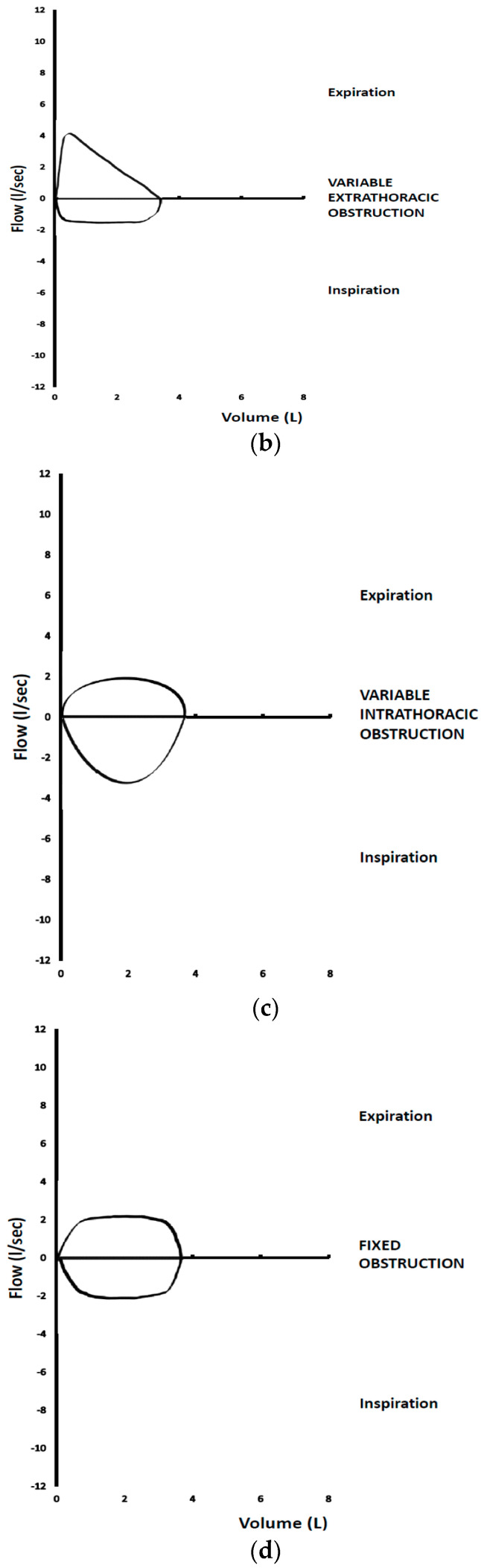

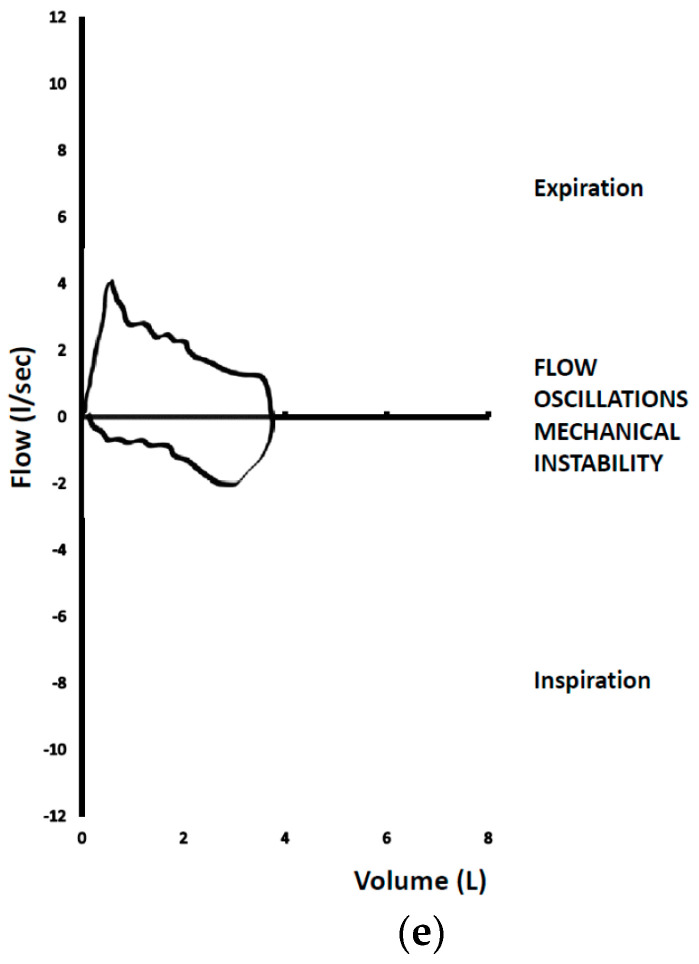
Common spirometric flow-volume loop patterns in patients with CAO. (**a**) Normal, (**b**) Variable Extrathoracic Obstruction, (**c**) Variable Intrathoracic Obstruction, (**d**) Fixed Obstruction, (**e**) Flow Oscillations/Mechanical Instability.

Flow-volume loop patterns (Figure 1a–e) have been assessed in the study by Raposo et al. and were also correlated with some quantitative criteria. Their findings confirmed these basic patterns, though they commented that the sensitivity is significantly lower than the one provided by the quantitative criteria [16].

The f-v loops alone are not sensitive enough to identify CAO but seem to have acceptably good specificity. Further to that, the f-v loops seem to associate relatively well with the location of the obstruction, and the accuracy improves when correlated with the quantitative criteria for identifying obstructions in the lower two-thirds of the trachea and in the right main bronchus [16].

The issue of low sensitivity, regarding the f-v loops, in identifying CAO has been addressed in a study by Modrykamien et al. [22]. The sensitivity was estimated at 5.5% for detecting CAO. Moreover, the positive predictive value was only 6.8% when the morphological criteria of plateau, biphasic curve, and oscillation features were evaluated.

Sensitivity would improve if a morphological criterion could be supported by the use of a quantitative criterion. Gelb et al., in a study assessing post-interventional changes in tracheal stenosis in COPD patients, found that the coexistence of multilevel obstruction [central and peripheral] can produce atypical f-v curves [49]. Le-Khac et al., in reviewing a small number of case series, found that external compression-induced CAO, due to goiter or upper mediastinal masses, might not be detectable when measurements are processed in a standing position but might be more pronounced when the f-v loops are obtained with the patients in a supine position [19].

Majid et al. tried to evaluate the role of f-v curves in cases of CAO, due to tracheobronchomalacia, by comparing them with the quantitative criteria on spirometry [20]. They found that a substantial number of patients had normal PFT values, despite the presence of moderate to severe tracheobronchomalacia, and that the f-v loops could also be inconclusive as well.

In another study by Sterner et al., which focused on the evaluation of the inspiratory curves in identifying intrathoracic or extrathoracic upper airway obstruction, the findings showed that if an inspiratory f-v loop is abnormal on at least one out of three efforts but the spirometric measurements remain normal, it is advisable to proceed to further investigation for possible CAO [18]. Although only 4.6% of the inspiratory curves were suggestive of CAO, 56% of them had abnormal features in at least two efforts. A cause was identified for 52% of them, with the most prevalent being vocal cord dysfunction. Other disorders related to the CAO findings were vocal cord paralysis, OSA, and neuromuscular disorders.

The overall message reflected throughout the literature is that flow-volume (f-v) loops are not a reliable way to safely rule out CAO. The inspiratory—expiratory curves are effort-dependent and the technicalities involving the patient, the operator, and the devices can provide variable results. For the obstruction to be detectable, the stenosis—fixed or variable—needs to be significant (usually more than 70%). The literature suggests that the most appropriate way to approach the qualitative criteria and the f-v loops would be to combine them with the quantitative criteria. This is well explained by Raposo et al., who managed to provide clarity on how to utilize the curves’ features and improve their sensitivity and specificity by interpreting them in tandem with the quantitative parameters [16].

## 11. The Role of Body Box Plethysmography

Whole body plethysmography appears to be very useful when it comes to the evaluation of airway resistance and compliance of the respiratory system. When CAO is primarily related to upper airway obstruction, the specific airway resistance loop has an S-deformation [52].

Gabathuler and Bühlmann suggested that CAO can be correlated with an impaired pattern of respiration that generates airway resistance above 6 cm H_2_O·L^−1^·s^−1^ during inspiration and/or expiration on spontaneous breathing [53]. Jamaati et al. found that plethysmography variables can be directly correlated with both the intensity and the length of stricture [54]. The intensity of the stricture had the strongest correlation with changes in upper airway resistance. Regarding the length of the stricture, plethysmography managed to identify some correlation, through the multivariate analysis, the reserve volume, and the total lung capacity.

## 12. Volume-Pressure Curves and Compliance

Volume-pressure curves are not easily and reliably obtained on spontaneous breathing and thus CAO cannot accurately be identified. Nouraei et al. tried to measure chest compliance changes and correlate them with the presence of CAO. Measurements were processed under general anesthesia, with a laryngeal mask to secure the airway, to eliminate confounding factors from the muscles and the elastic properties of the chest wall [48]. The findings showed that pulmonary compliance, on spontaneous non-forceful breathing, was strongly correlated with the severity of the anatomical stenosis. The correlation was more accurate and representative of the degree of stenosis when compared to the measurements obtained by spirometry.

The importance of the above finding lies in the rationale that activties individuals do not normally perform forced inspiratory/expiratory maneuvers in daily activities, as they would in spirometry. This implies that compliance changes, when a stenosis is present, might be more indicative of the underlying pathology and become better correlated with the clinical presentation if measurements are obtained on normal tidal breathing.

## 13. Oscillometry

Another modality that has been utilized to detect CAO is oscillometry. Impulse oscillometry has been used in identifying an element of vocal cord dysfunction [VCD] in the upper airways, by assessing changes in Zrs on tidal breathing and comparing Rrs in inspiration and expiration [55,56].

The Zrs can reflect large airway swings in inspiration, indicating VCD. In a similar way, the difference of Rrs during the inspiratory and expiratory phase can demonstrate abnormal behaviour of the glottis implying VCD.

Accordingly, oscillometry in upper airway assessments has been utilized in obstructive sleep apnoea [OSA] as well. In a study by Cao et al., Zrs, Rrs 5 and Rrs 20 were utilized to assess the effect of body posture in individuals with suspected OSA. The measurements showed that OSA cases had increased Zrs and Rrs in a supine position, indicating a positive predictive value for OSA [57].

Measurements during the use of C-PAP, for the treatment of OSA, have shown improvement in Rrs, in the inspiratory and expiratory phase, by the implementation of positive airway pressures through C-PAP [58].

Regarding the use of oscillometry in the evaluation of other types of CAO, it seems that its main advantage over the conventional PFTs applies in two cases: in CAO that coexists with peripheral airway disease and in demonstrating the post-intervention improvement of resistances and flows.

Verbanck et al. proposed a model of approach that suggests a relatively accurate way to identify CAO being unaffected by peripheral airway obstruction [59]. The study introduced a new index, the DR/DV ratio, which stands for difference in resistance [DR] over difference in flow [DV]. They initially validated the findings for the peripheral airways in both normal and COPD cases and found that the ratio was unaffected by the peripheral airflow limitation. On applying the ratio to different flow rates, up to 1 L/s, it was demonstrated that resistance changes are flow-dependent for CAO and in particular for tracheal stenosis with diameter 42 ± 28 mm. Both the Rrs and the ratio are higher on tracheal stenosis and improve/decrease significantly after dilatation of the stenotic area. Yassuo et al. showed that the difference in resonance frequency [ΔFres] had the best AUC value in distinguishing CAO from COPD and in particular from the phenotype with airway wall thickening. The findings support that ΔFres can become a reliable indicator in discriminating between CAO and peripheral airflow limitation [50].

As far as the CAO and the relevant post-intervention changes are concerned, oscillometry can demonstrate them in a reliable way. Oscillometry measurements, in the presence of CAO, had relatively higher values in R5 and R20, Fres, and ALX and a negative reactance at 5 Hz [60]. After dilation was accomplished, through interventional bronchoscopy, a considerable change of the Rrs 20 was identified, suggesting that it could reliably evaluate mechanical changes in CAO. Handa et al. also used oscillometry before and after interventions for fixed and variable/dynamic CAO [3]. Their study showed that Rrs at 5 and 20 Hz and Xrs at 5 Hz are valuable indices in discriminating fixed and variable CAO.

In variable CAO, the Rrs were markedly frequency-dependent, while in the fixed CAO, the Rrs were frequency-independent. Another finding of the study was that Rrs 5 and Rrs 20 had better sensitivity and specificity, when correlated to dyspnea scores, in comparison to the spirometric findings.

Oscillometry measurements were also used before and after bronchoscopic intervention for post-tracheostomy tracheal stenosis in patients with a history of neurological disorder [61]. Tracheal stenosis, with diameter between 2 to 13 mm, increased the Rrs and decreased the Xrs at low frequency and increased the Fres. After the intervention, the total Zrs decreased and Rrs 5 decreased in a similar way. When patterns of improvement were investigated with spirometry, the correlation was poor compared to the oscillometry findings.

## 14. Answering the Key Clinical Questions and Limitations

The data presented might seem somehow controversial. Despite the fact that they demonstrate the usefulness of PFTs in diagnosing the CAO, by enlightening different relevant aspects, there is no unified approach. The many different indices and parameters can only answer one part of the clinical scenario at a time. Revisiting the key clinical questions, addressed initially, might bring more clarity on how the quantitative and qualitative criteria can be utilized.

Table 2 summarizes the utility of PFTs in CAO and their clinical relevance.

Starting with the level of stenosis, the most useful indices for the upper central airway are the EDI, f-v curves (combined with the quantitative criteria), and oscillometry. The CAO of the trachea and main bronchi is better revealed by using FEF50%/FIF50% and Empey index.

The wall region involved is not well described in the literature and none of the criteria is reliable to safely answer that.

The variable obstructions are better demonstrated with oscillometry, especially when the upper airway is involved. The f-v curve is quite indicative of fixed CAO—intra or extra thoracic—when the shape gives the typical flattening in both the inspiratory and expiratory phase.

The severity of the stenosis is not an easy question to answer. As demonstrated in Table 2, different indices have been used in different studies and populations.

The most interesting data are related to the identification of improvement after an intervention—for severe CAO—and the coexistence of peripheral airway obstruction. In both cases, oscillometry has shown promising data that are easier to reproduce with more consistency.

The difficulty in providing straightforward answers to the above clinical questions demonstrates the clinical complexity of CAO and the relevant challenges through PFTs. In comparison to other modalities used, such as dynamic CT imaging and bronchoscopy—which remain the gold standard of diagnosis—PFTs are effort-dependent. This means that when the individuals are trying to perform them, the outcome is very much dependent on the quality of their effort. The repeatability is a key parameter and is related to a variety of factors. The fact that, in some studies, oscillometry provides better results might be linked to the fact that, as a modality, oscillometry is less effort-dependent.

## 15. Conclusions

Pulmonary function tests, including spirometry, plethysmography, and oscillometry, can provide a useful tool in identifying some features of CAO and alert the clinician to carry out further investigations. Clinically apparent central airway obstruction is characterized by significant morbidity. PFTs can be utilized as an introductory modality in approaching a case with suspected CAO and become a good way of following up in cases where intervention improved a clinically significant CAO. Their role in the initial assessment, but more importantly in monitoring high risk patients with diagnosed or partially improved CAO, could be helpful but is still questionable. PFTs, including oscillometry, have been demonstrated to have low-moderate sensitivity as screening modalities for airway obstruction and cannot be relied upon in the absence of other modalities such as bronchoscopy and CT scans. Nevertheless, they may be used as an added data point when dealing with severe airway obstruction in various locations. More research is necessary to determine whether treated airway obstruction can demonstrate improvement in the parameters mentioned.

## Figures and Tables

**Table 1:** Sensitivity and specificity of qualitative and quantitative spirometric parameters in different studies.

Study Authors	Study Details	Objective of Study	Sensitivity	Specificity	Main Conclusion
Raposo et al. [16]	82 patients assessed with PFTs and bronchoscopy; Clinicians assessed f-v curves, being blind for the quantitative criteria	f-v curves and correlation with quantitative parameters; accuracy in identifying CAO	f-v curves alone; 30.6% quantitative criteria alone; 88.9% f-v curves combined with quantitative criteria; 93.9%	f-v curves alone; 91.3% quantitative criteria alone; 93.5% f-v curves combined with quantitative criteria; 89.8%	Sensitivity and specificity improve by combining qualitative with quantitative criteria FEF50%/FIF50% ≥ 1 is the most accurate quantitative criterion in identifying location of CAO Empey index ≥ 10 is the most accurate quantitative criterion in identifying extraluminal and mixed obstruction f-v curves are better in identifying intraluminal obstruction FEF50%/FIF50% ≥ 1 and Empey index ≥ 8 are more accurate in identifying the degree of stenosis
Miller et al. [21]	144 patients with goiter assessed with f-v curves; 44 patients were identified with possible CAO Further assessment with f-v curves post-intervention	Correlation of f-v curves with suspected external CAO due to goiter Empey index also assessed	f-v curves; 100% Empey index ≥ 8; 64%	f-v curves; 78% Empey index ≥ 8; 94%	f-v curves are sensitive in identifying external CAO due to goiter
Modrykamien et al. [22]	995 PFTs assessed regarding qualitative criteria [f-v curves] and quantitative criteria [Empey index ≥ 10, FEF50%/FIF50% < 0.3 or >1, PIF < 100, FEV1/FEV0.5 > 1.5]; 475 f-v curves met inclusion quality criteria	Evaluation of f-v curves and quantitative parameters in accurately identifying CAO/UAO	f-v curves; 5.5% Empey index ≥ 10; 8.3% FEF50%/FIF50% < 0.3 or >1; 47.2% PIF < 100; 8.3% FEV1/FEV0.5 >1.5; 30.5%	f-v curves; 93.8% Empey index ≥ 10; 96.8% FEF50%/FIF50% < 0.3 or >1; 55.2% PIF < 100; 91.1% FEV1/FEV0.5 >1.5; 60.5%	f-v curves have low sensitivity in detecting CAO/UAO Quantitative criteria have better sensitivity than the f-v curves but still not good enough in accurately identifying CAO/UAO
Nouraei et al. [23]	9357 PFTs from normal and non-UAO individuals and 217 PFTs from individuals with CAO due to laryngotracheal stenosis	Evaluation of the EDI in differentiating laryngotracheal stenosis from other diagnosis	EDI > 50; 95.9%	EDI > 50; 94.2%	EDI can reliably identify CAO due to tracheolaryngeal stenosis
Schuering et al. [24]	50 patients with subglottic stenosis and 32 asthma patients were included	Evaluation of the EDI in accurately identifying CAO due to subglottic stenosis vs. asthma patients	EDI > 48; 88%	EDI > 48; 84.4%	EDI has good diagnostic accuracy in differentiating CAO due to subglottic stenosis from asthma patients
Calamari et al. [25]	44 patients with UAO and 895 patients without UAO compared regarding the EDI	Evaluation of the EDI regarding its ability to identify UAO when body habitus/BMI is taken into consideration	EDI > 50 Non-obese; 83.3% EDI > 50 Obese; 50%	EDI > 50 Non-obese; 56.2% EDI > 50 Obese; 71.9%	EDI has low sensitivity in obese patients in identifying UAO but has better specificity EDI alone is not a reliable criterion in accurately identifying UAO in obese patients
Ntouniadakis et al. [26]	43 asthma patients, 31 COPD patients and 50 patients with subglottic stenosis were assessed.	Evaluation of spirometry parameters—with a specific focus on the EDI—and of the dyspnea index in accurately identifying CAO due to subglottic stenosis when compared to cases with peripheral airway obstruction due to asthma and COPD	EDI > 39; 98%	EDI > 39; 96%	Increased dyspnea index when combined with EDI > 39 can raise suspicion for subglottic stenosis and justify further investigation with endoscopic evaluation
Garcia-Pachon et al. [27]	54 UAO patients, 60 peripheral chronic airflow limitation [CAL] patients and 23 with mixed UAO and CAL patients assessed with PFTs focusing on the following parameters; FIF50%, FEF50%/FIF50% ≥ 1, Empey Index ≥ 10, FEV1/FEV0.5 ≥ 1.5, MVV/FEV1 < 25	Evaluation of several PFT parameters in accurately identifying UAO in patients that also have peripheral airway obstruction	Best values with: FEF50%/FIF50%; UAO alone; 85% UAO + CAL; 35% Empey index; UAO alone; 72% UAO + CAL; 52% MVV/FEV1; UAO alone; 74% UAO + CAL; 56%	Best values with: FEF50%/FIF50%; UAO alone; 100% UAO + CAL; 100% Empey index; UAO alone; 93% UAO + CAL; 93% MVV/FEV1; UAO alone; 100% UAO + CAL; 100%	Sensitivity of the several indices is low when UAO is combined with CAL When at least 3 indices are combined the presence of UAO, in patients with CAL, can be suspected and justify further investigation The most specific criterion in all cases is MVV/FEV1

**Table 2:** Utility of PFTs in CAO and their clinical relevance.

Clinical Question	PFT	Type of Correlation
Level of stenosis [intra vs. extra thoracic]	FEF50%/FIF50%	Any part of the airway excluding mid-trachea
	Empey index	Lower 1/3 of trachea
	EDI	UAO
	PIF	Extrathoracic
	PEF	Subglottic stenosis
	MVV/FEV1	UAO
	F-V curves	Lower 2/3 of trachea and RMB
		UAO
	Body box plethysmography	UAO
	Oscillometry	UAO (OSA, VCD)
		Tracheal stenosis
Wall region involved [cartilaginous vs. membranous]	Non-sensitive or specific	-
Fixed vs. Variable	Empey index	Fixed external extrathoracic obstruction
	EDI	Variable obstruction, primarily at BMI > 30
	F-V curve	Flattened inspiratory and expiratory phase
	Oscillometry	Variable obstruction, primarily of UAO
Severity of stenosis	FEF50%/FIF50%	≥1, when correlated with f-v curves
	Empey index	≥8 mL/L/min
	EDI	≥75
	Body box plethysmography	Severe strictures increase upper airway resistance
	V-P curves and Compliance	Severity of stricture when measured intra-operatively with closed circuit
	F-V curves	If CAO is ≥ 70%
	PEF	Severe tracheal stenosis
	Oscillometry	Increased DR/DV, Rrs when tracheal stenosis
		Increased Zrs, Rrs in supine position in OSA
Identify improvement of CAO after intervention	FEF50%/FIF50%	decrease
	Empey index	decrease
	EDI	decrease
	PEF	increase
	V-P curves and Compliance	increase
	Oscillometry	Decrease of DR/DV, Rrs, Fres
		Increase of Xrs
Identify CAO when coexistent with peripheral airway disease	EDI	≥48 when asthma is present
	MVV/FEV1	<25 when UAO with peripheral obstruction
	Oscillometry	DR/DV, ΔFres
